# How contraction has shaped evolution

**DOI:** 10.7554/eLife.52805

**Published:** 2019-11-14

**Authors:** Mukund Thattai

**Affiliations:** Simons Centre for the Study of Living MachinesNational Centre for Biological Sciences, Tata Institute of Fundamental ResearchBangaloreIndia

**Keywords:** cellularization, coenocyte, evolution, epithelium, actin, multicellularity, Other

## Abstract

Two unicellular relatives of animals reveal that coordinated contractions of groups of cells using actomyosin predated animal multicellularity during evolution.

**Related research article** Dudin O, Ondracka A, Grau-Bové X, Haraldsen AA, Toyoda A, Suga H, Brate J, Ruiz-Trillo I. 2019. A unicellular relative of animals generates an epithelium-like cell layer by actomyosin-dependent cellularization. *eLife* **8**:e49801. doi: 10.7554/eLife.49801

The Cheshire Cat in *Alice’s Adventures in Wonderland* is famous for its disappearing act: parts of its body vanish one by one until nothing remains but its ethereal grin. Scientists attempting to retrace the evolution of animals confront something equally curious. One might assume that going ever further back in evolutionary time, recently-evolved animal traits would drop away until a sort of ‘minimal animal’ remained. However, a growing body of data suggests that this minimal animal may not be an animal at all. Instead, sophisticated cellular processes once thought to be exclusive to animals are found across several unicellular eukaryotes: grins without (multicellular) cats!

So how do scientists reconstruct the deep history of multicellularity? The record for the oldest multicellular organism to be studied directly belongs to plants grown from 30,000-year-old seeds preserved in permafrost (Yashina et al., 2012). However, multicellular life is much older than this. Fossils of multicellular red algae have been dated to 1.6 billion years ago (Bengtson et al., 2017), and fossils of multicellular fungi date from about a billion years ago (Loron et al., 2019). The oldest confidently-dated animal fossils are about half a billion years old (Bobrovskiy et al., 2018). Multicellularity arose independently in plants, fungi and animals (Brunet and King, 2017). Scientists are interested in the unicellular ancestors of these groups because they want to know if each transition into multicellularity was driven by similar evolutionary forces. Unfortunately, fossils reveal little about the cell biology of these primordial organisms.

Darwin was well aware of this challenge. To reconstruct the evolutionary history of an organism, he wrote in *On the Origin of Species*, “we ought to look exclusively to its lineal ancestors; but this is scarcely ever possible and we are forced in each case to look to… the collateral descendants from the same original parent-form.” That is, one must hope the traits of surviving organisms reveal those of their extinct ancestors. Researchers now know that Darwin’s idea of “living fossils” was too simplistic. No organism remains entirely identical to its ancestor: genetic mutations constantly accumulate, driven by conflict, competition, and random chance. Nevertheless, one could hope to reconstruct the ancestor using a patchwork of different ancestral traits preserved across different surviving descendants.

A central theme of the emerging field of evolutionary cell biology is to study organisms that provide as much information as possible about the past. One way to do this is to develop new model organisms based on their position in the tree of life. As the evolution of animals is retraced, an ancestral unicellular species at the very threshold of multicellularity will eventually be reached. It is possible this species has no surviving descendants, other than the animals themselves. To find more collateral descendants, one must push further back in time. The better life’s existing diversity is sampled, the more likely that a species will be found similar to the ancestors scientists want to reconstruct.

The billion-year-old clade known as Holozoa consists of animals and closely related unicellular species, including choanoflagellates, filastereans, and ichthyosporeans. Just a decade ago this was a sparsely sampled region of the eukaryotic tree: for example, the first choanoflagellate genome was only published in 2008 (Brunet and King, 2017). Today dozens of holozoan species have been cultured, sequenced, and studied, and they are a fertile hunting ground for interesting cell biology. Importantly, the non-animal holozoans include species that can become transiently multicellular, at certain times or under certain conditions. Specifically, some choanoflagellates and ichthyosporeans have clonal multicellular life stages, while some filastereans form multicellular aggregates. But are these behaviors homologous to multicellularity in animals, and therefore representative of the ancestral state? Or are they examples of convergent evolution, driven by adaptations to similar environments?

One way to answer these questions is to resolve the molecular mechanisms that enable multicellular behavior across holozoans. Suggestively, holozoan genomes encode transcription factors and cell adhesion genes known to be essential for animal multicellularity, but the roles of these genes had not been directly demonstrated (Grau-Bové et al., 2017; Richter et al., 2018). Now, two independent teams have reported the results of studies on certain animal-like behaviors in unicellular lineages that shed light on the evolution of animal multicellularity (Figure 1).

**Figure 1.**
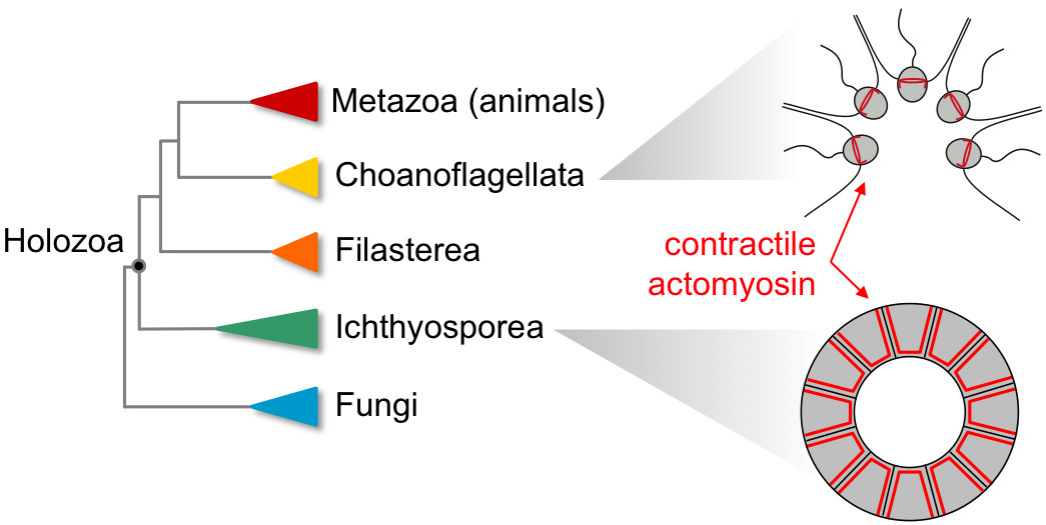
Non-animal species in the clade Holozoa exhibit coordinated contractions dependent on actomyosin complexes similar to those observed in modern animals. Phylogenetic tree of the Holozoa showing the position of animals (Metazoa), choanoflagellates (Choanoflagellata), filastereans (Filasterea) and ichthyosporeans (Ichthyosporea) within this clade (left). The choanoflagellates include *Choanoeca flexa* (top right), which has flagella that point inwards when the organism is in bright light. In the dark, the cells contract in a coordinated manner that causes the flagella to point outwards, a movement reminiscent of the contractions that cause tissues to curve during animal development (figure adapted from Brunet et al., 2019). The ichthyosporean *Sphaeroforma arctica* (bottom right) also exhibits actomyosin contractility, invaginating its membrane to generate multiple cells out of a polarized epithelial layer. This movement is comparable to processes that occur during embryonic development in flies (Dudin et al., 2019).

In a paper in eLife, Iñaki Ruiz-Trillo and co-workers from Barcelona, Liverpool, Oslo, Shizuoka and Hiroshima – including Omaya Dudin and Andrej Ondracka as joint first authors – report how reproduction in an ichthyosporean called *Sphaeroforma arctica* involves a stage of growth that is reminiscent of the embryonic development of fruit flies. The nucleus of an initial single cell divides repeatedly to form a polarized epithelial layer, which then gives rise to multiple cells as its membrane undergoes coordinated invaginations (Dudin et al., 2019).

In a second paper in Science, Nicole King and co-workers from Berkeley and Amsterdam – including Thibaut Brunet, Ben Larson and Tess Linden as joint first authors – report the results of a study on a newly isolated choanoflagellate which they name *Choanoeca flexa* (Brunet et al., 2019). In bright light this organism exists as a cup-shaped colony of cells, with their flagella pointing inwards. In the dark, however, the cup flips inside-out via a collective cellular contraction. This collective contraction is reminiscent of the contractions that generate curvature in developing animal tissues.

Both studies use imaging and pharmacological inhibition to demonstrate that these multicellular processes depend on the same molecular machinery: complexes of actin and myosin that can generate mechanical forces within cells. These results suggest that the last common ancestor of holozoans was an organism that was capable of transient multicellularity, with cells that could contract collectively. Among its descendants, only the animals evolved a permanently multicellular lifestyle, using the power of collective contraction to sculpt tissues and generate the “endless forms most beautiful” that so inspired Darwin.
